# Advances in Sn-Based Catalysts for Electrochemical CO_2_ Reduction

**DOI:** 10.1007/s40820-019-0293-x

**Published:** 2019-07-29

**Authors:** Shulin Zhao, Sheng Li, Tao Guo, Shuaishuai Zhang, Jing Wang, Yuping Wu, Yuhui Chen

**Affiliations:** State Key Laboratory of Materials-oriented Chemical Engineering, School of Energy Science and Engineering, Nanjing Tech University, Nanjing, 211816 Jiangsu People’s Republic of China

**Keywords:** Greenhouse effect, CO_2_ electrochemical reduction, Sn-based electrocatalysts

## Abstract

The increasing concentration of CO_2_ in the atmosphere has led to the greenhouse effect, which greatly affects the climate and the ecological balance of nature. Therefore, converting CO_2_ into renewable fuels via clean and economical chemical processes has become a great concern for scientists. Electrocatalytic CO_2_ conversion is a prospective path toward carbon cycling. Among the different electrocatalysts, Sn-based electrocatalysts have been demonstrated as promising catalysts for CO_2_ electroreduction, producing formate and CO, which are important industrial chemicals. In this review, various Sn-based electrocatalysts are comprehensively summarized in terms of synthesis, catalytic performance, and reaction mechanisms for CO_2_ electroreduction. Finally, we concisely discuss the current challenges and opportunities of Sn-based electrocatalysts.

## Highlights

- This review summarizes current developments in the fabrication of tin (Sn)-based electrocatalysts for CO_2_ reduction.
- Sn-based electrocatalysts are comprehensively summarized in terms of synthesis, catalytic performance, and reaction mechanisms for CO_2_ electroreduction.
- The remaining challenges and opportunities for Sn-based electrocatalysts in the field of CO_2_ electroreduction are briefly proposed and discussed.

## Graphical abstract

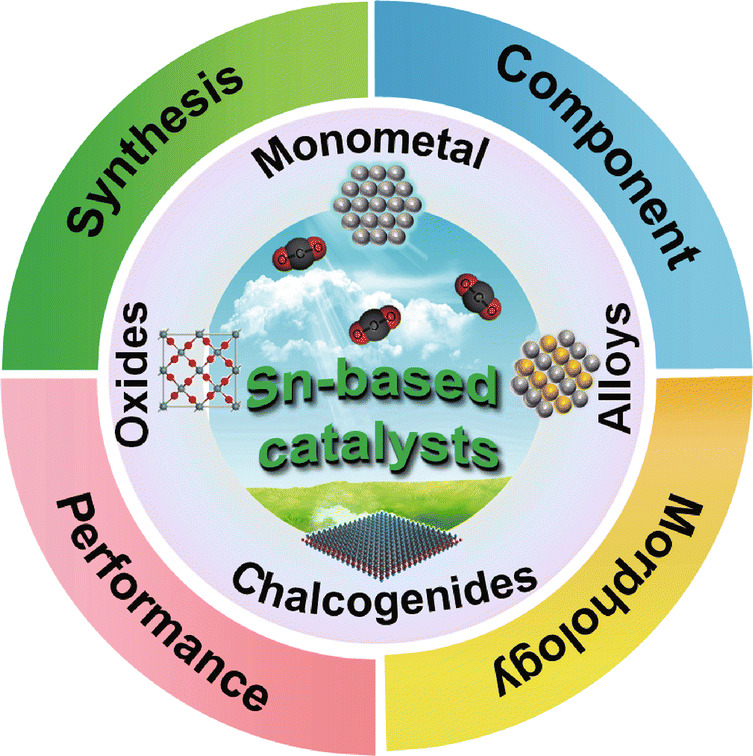

## Introduction

The excessive dependence on fossil fuels in the past has created an energy crisis, and a large quantity of carbon dioxide (CO_2_) has been released into the atmosphere, which is responsible for global warming [1, 2]. This inspired us to develop efficient methods to reduce CO_2_ emissions and to convert CO_2_ into value-added chemicals, which would not only mitigate the high atmospheric CO_2_ concentration but also produce renewable fuels to relieve the shortage of energy [3]. At present, some CO_2_ conversion technologies, including enzymatic, photocatalytic, thermocatalytic, as well as electrochemical reduction, have been developed to convert CO_2_ into useful fuels and chemicals [4–8]. Among these technologies, the electrochemical reduction of CO_2_ (CO_2_ER) is preferred owing to its three advantages: First, it can be conducted under milder conditions, such as ambient pressure and temperature, compared with conventional industrial processes. Second, its products can be customized by manipulating reaction parameters (e.g., electrocatalysts, electrolyte, and redox potential). Third, the CO_2_ER can completely utilize intermittent electricity such as wind electricity and solar electricity.

Generally, CO_2_ can be converted to various products, such as formic acid (HCOOH), carbon monoxide (CO), methane (CH_4_), methanol (CH_3_OH), and ethanol (C_2_H_5_OH), via different pathways and half-reactions, as shown in Table 1 [9]. Of these, formate (HCOOH and HCOO^−^) is a basic organic chemical raw material that can be used as fuel in a direct formate fuel cell, as a means of H_2_ storage, and as feedstock in the synthesis of fine chemicals that are of interest to the pharmaceutical industry [10]. Additionally, Sargent and coworkers evaluated the economic viability of various chemicals from the CO_2_ER and they found that formic acid has great business value [11]. Thus, formate is one of the most desired products. In addition to formate, CO is another major product produced during the process of CO_2_ER, and it is easy to separate from solution and can be further converted to hydrocarbons through the Fischer–Tropsch process [12]. Although the theoretical potentials, as shown in Table 1, required to form target products are not negative, more negative potentials must be applied in practical reactions because of complicated reaction mechanisms and sluggish kinetics. The overpotential of each step must be overcome, and one of those steps could have a large overpotential that makes the overall reaction sluggish [13, 14]. The high reduction overpotential leads to a waste of energy and significant H_2_ evolution reaction (HER), which is a major side reaction that prevails over the CO_2_ER [15]. Therefore, the exploitation of an electrocatalyst with high activity and selectivity is highly desirable for expediting reaction kinetics and efficiency.

**Table 1:** Electrochemical potentials of several possible CO_2_ reduction reactions in aqueous solutions

CO_2_ reduction half-reactions	Electrode potentials (*V* vs. RHE) at pH = 7
CO_2_ (g) + 2H^+^ + 2e^−^ → CO (g) + H_2_O (l)	− 0.106
CO_2_ (g) + 2H^+^ + 2e^−^ → HCOOH (l)	− 0.250
CO_2_ (g) + 4H^+^ + 2e^−^ → HCHO (l) + H_2_O (l)	− 0.070
CO_2_ (g) + 6H^+^ + 6e^−^ → CH_3_OH (l) + H_2_O (l)	0.016
CO_2_ (g) + 8H^+^ + 8e^−^ → CH_4_ (l) + 2H_2_O (l)	0.169
2CO_2_ (g) + 12H^+^ + 12e^−^ → C_2_H_4_ (g) + 4H_2_O (l)	0.064
2CO_2_ (g) + 12H^+^ + 12e^−^ → C_2_H_5_OH (l) + 2H_2_O (l)	0.084

CO_2_ electrocatalysts, including Au, Ag, Pd, Cu, Sn, and their related metal oxide and carbon nanocomposites, have been widely used in the CO_2_ER to produce formate and CO. Among them, Sn-based catalysts have emerged as an interesting metal for their catalytic power, selectivity to formate, and their non-noble, eco-friendly, and low-cost characteristics [16–18]. Until now, various Sn-based catalysts including single metals, alloys, oxides, sulfides, and their hybrids with carbon nanomaterials (e.g., carbon nanotubes and graphene) or metal oxide have been reported for the CO_2_ER. At present, the Faradaic efficiency (FE) of formate and CO can reach ~ 100% and over 90%, respectively [19–21]. Moreover, the catalyst types, sizes, morphologies, surface modification, and reaction conditions exhibit excellent effects on the performance of CO_2_ER. Until now, many excellent reviews have focused on the preparation and applications of metal-based CO_2_ electrocatalysts [3, 22–26]. However, most of them are broad and comprehensive summaries. Therefore, there is a great need to provide a timely and specific overview of Sn-based catalysts for the CO_2_ER.

In this review, we first discuss the representative reaction pathways of Sn-based electrocatalysts, followed by summarizing the recent progress of Sn-based heterogeneous CO_2_ER electrocatalysts. All Sn-involved catalysts can be classified into four categories: monometallic Sn, bimetallic or multimetallic Sn, Sn oxides, and Sn sulfides. For each category, we provide examples of catalysts, including the description of their preparation process, catalytic activity and products, and strategies for improving its performance. Finally, we briefly propose and discuss the challenges and opportunities in this field.

## Reaction Mechanism and Pathways of the Sn-Based Electrocatalysts

**Fig. 1:**
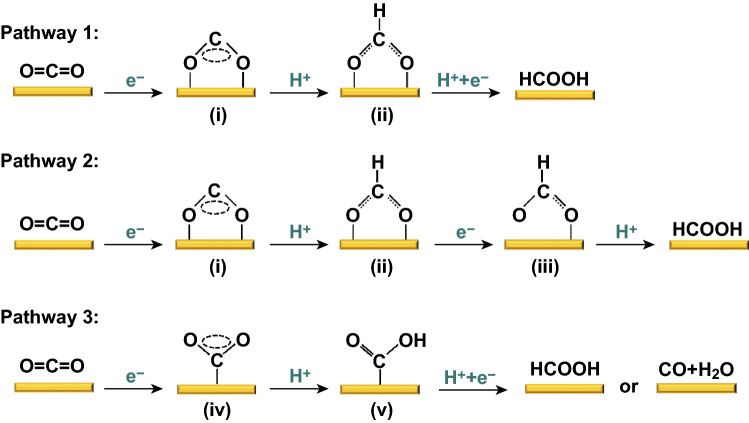
Possible reaction pathways for electrocatalytic CO_2_ER on the Sn-based catalysts in aqueous solutions

Sn-based catalysts have high selectivity in forming formate and CO, and the reaction mechanism of the formate and CO pathways are relatively simple compared to other pathways. To date, although many works have experimentally demonstrated Sn-based electrocatalysts to be an effective kind of catalyst for reducing CO_2_ to formate and CO, the underpinned mechanisms are not yet fully understood. Generally, there are three steps involved in the generation of products, in theory: (1) adsorption of the reactant on the surface of the electrocatalyst; (2) electron/proton transfer to the reactant; and (3) desorption of the products from the electrocatalyst surface. A variety of approaches, including computational [27, 28], electrokinetic [29], and in situ analysis [30], have been proposed to study the reaction mechanism. The selectivity for a catalyst, which is largely determined by the first proton coupling, takes place at the C or O of CO_2_^·−^. As shown in Fig. 1, based on some prevalent viewpoints, the formation of formate and CO goes through the following three pathways. Pathway 1: CO_2_^·−^ radical anion (i) is first formed via a one-electron transfer to CO_2_ [18, 31], where the oxygens in the CO_2_^·−^ radical anion (i) are bound to the electrode surface. In this case, the protonation takes place on the carbon atom and forms an HCOO^·^ intermediate (ii), and then, a second electron transfer and protonation step result in the HCOOH product [32]. Pathway 2: unlike pathway 1, theoretical calculations propose that ^·^OCHO intermediates (iii) can be formed after the HCOO^·^ intermediate (ii) via an electron transfer [33]. After that, HCOOH production occurs via the ^·^OCHO (iii) protonation step. Pathway 3: when the carbon in CO_2_^·−^ is bonded to the electrode surface (iv), the CO_2_^·−^ may also be reduced via the protonation of its oxygen atom, resulting in the formation of ^·^COOH (v). This intermediate is then either reduced to HCOOH or loses H_2_O to form CO [34].

A mechanism is closely related to the types of catalysts and environments; therefore, theoretical researchers can design catalysts to manipulate the reaction mechanism in the desired pathways. For example, Wallace et al. developed Sn-modified N-doped porous carbon nanofiber (Sn-CF) catalysts for CO_2_ electroreduction [21]. Two kinds of catalysts were synthesized in which the Sn species existed in different forms. The first catalyst consisted of Sn nanoparticles (NPs) covering the nanofiber surface (Sn-CF1000) and had high FE for formate; the other one was simply atomically dispersed Sn in an N-doped carbon nanofiber catalyst (AD-Sn/N-C1000) that drove efficient CO formation. For the Sn-CF1000, the slope of the Tafel curve was 79 mV dec^−1^, close to the theoretical value of 59 mV dec^−1^ for a rapid one-electron transfer step followed by a rate-determining step (RDS). The result suggested that Sn-CF1000 could bind the CO_2_^· –^ intermediates strongly. This might account for the increased formate formation at a relatively low overpotential. The AD-Sn/N-C1000 exhibited a Tafel slope of 140 mV dec^−1^, which is close to the theoretical value of 120 mV dec^−1^ for a mechanism in which the initial single-electron transfer forming CO_2_^· –^ intermediates is the rate-determining step for CO_2_ to CO conversion. The improved activities for CO formation on AD-Sn/N-C1000 may be mainly attributed to the enhanced stabilization of CO_2_^· –^ and subsequently facilitate the formation of ^·^COOH intermediates on the Sn–N moieties. In addition, the local pH has great influence on the mechanism. It has been reported that high current density means a higher consuming rate of the proton source, which leads to a significant increase in the local pH value (making the local pH alkaline) near the catalytic sites. A higher local pH leads to an increase in CO selectivity, or the formation of other C1–C2 products through reaction intermediates, such as CO. Herein, we take SnO/C as an example. Hu’s group reported that densely packed ultra-small SnO NPs could enhance CO_2_^· –^ absorption and increased the local pH, and demonstrated that the local pH increase suppressed formate formation, while it had less influence on the formation rate of CO [35].

**Fig. 2:**
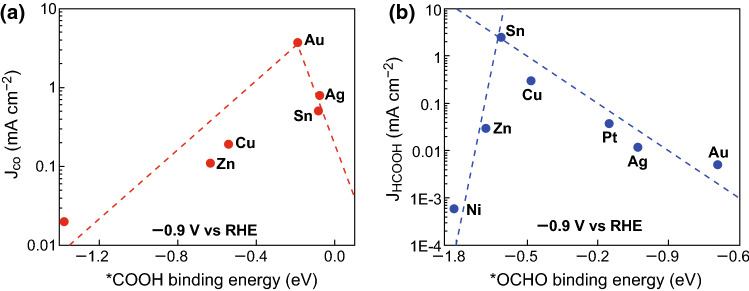
**a** Volcano plot using ^·^COOH binding energy as a descriptor for CO partial current density at − 0.9 V (vs. RHE). **b** Volcano plot using ^·^OCHO binding energy as a descriptor for HCOO^−^ partial current density at − 0.9 V (vs. RHE). Reprinted with permission from Ref. [33]

Jaramillo et al. combined an experiment with the theoretical investigation of the CO_2_ER to HCOO^−^ on polycrystalline Sn surfaces to understand the mechanism and key intermediates for HCOO^−^ production [33]. They focused on gaining insight by comparing the CO and HCOO^−^ production of Sn electrodes to other polycrystalline metal foil catalysts (e.g., Au, Ag, Cu, Zn, Pt, and Ni) at − 0.9 V versus the reversible hydrogen electrode (RHE) . For CO production, each metal’s partial current densities while forming CO were plotted against the binding energies of ^·^COOH (v) on each metal surface. A clear volcano trend can be observed, and Sn appeared on the weak binding leg of the volcano, supporting the notion of ^·^COOH intermediate as a descriptor for CO production (Fig. 2a). For HCOO^−^ production, a clear volcano trend for partial current against binding energy of ^·^OCHO (iii) can be observed as well (Fig. 2b). Sn appeared near the top of this volcano, implying that Sn had a near-optimal binding energy for the key intermediate ^·^OCHO (iii) to produce HCOO^−^. This volcano suggests that ^·^OCHO is a key intermediate for HCOO^−^ production on transition metals, and it rationalizes the high selectivity toward HCOO^−^ of an Sn catalyst.

In addition to computational science and kinetic studies, in situ characterization techniques, such as IR and Raman analyses, were conducted to characterize the surface species at Sn interfaces when CO_2_ reduction was taking place. Bocarsly’s group applied attenuated total reflectance infrared (ATR-IR) spectroscopy in situ to study the mechanism of CO_2_ reduction on Sn films covered by SnO_*x*_ [30]. They found that the surface-bound monodentate Sn carbonate species was a crucial electroactive intermediate for transforming CO_2_ into HCOO^−^ at Sn electrodes. Broekmann et al. utilized potential- and time-dependent operando Raman spectroscopy to monitor the oxidation state changes of SnO_2_ that accompany CO_2_ reduction [36], and they established a correlation between the oxidation state of the SnO_2_ NPs and their FE for the production of formate. They found that the NPs exhibited a high FE in the production of formate at moderately cathodic potentials, while the oxide was reduced to metallic Sn at negative potentials, which led to a significant degradation in efficiency.

## Advanced Sn-Based Catalysts for Electrochemical CO_2_ Reduction

Recently, various Sn-based electrochemical CO_2_ reduction electrocatalysts have been studied intensively, including monometallic Sn catalysts, bimetallic or multimetallic Sn catalysts, Sn oxides, and Sn sulfides. Detailed CO_2_ER performances of Sn-based electrocatalysts are summarized in Table 2.

**Table 2:** Performance of Sn-based catalysts in CO_2_ electroreduction

Catalyst	FE (%)	Major products	Potential at FE_max_ (*V*)	Electrolyte	Current density (mA cm^−2^)	References
Sn rod	94	HCOOH	− 1.6 (vs. Ag/AgCl)	Pure water	–	[45]
Sn quantum sheets/GO	89	HCOO^−^	− 1.8 (vs. SCE)	0.1 M NaHCO_3_	–	[48]
Sn-CF1000	62	HCOO^−^	− 0.89 (vs. RHE)	0.1 M KHCO_3_	11	[21]
AD-Sn/N-C1000	91	CO	− 0.69 (vs. RHE)	0.1 M KHCO_3_	1.75	
Cu@Sn	100	HCOO^−^	− 0.93 (vs. RHE)	0.5 M NaHCO_3_	16.52	[19]
Cu_*x*_O–Sn nanowire	90	CO	− 0.8 (vs. RHE)	0.1 M KHCO_3_	4.5	[63]
Cu–Sn foams	93–94	CO	− 0.75 to − 0.9 (vs. RHE)	0.1 M KHCO_3_	6.2	[12]
Cu–Sn alloy	90	CO	− 0.6 (vs. RHE)	0.1 M KHCO_3_	1.0	[58]
CuSn_3_	95	HCOO^−^	− 0.5 (vs. RHE)	0.1 M KHCO_3_	33	[64]
Cu_0.2_Sn_0.8_	85	HCOOH	− 0.35 (vs. RHE)	0.5 M NaHCO_3_	–	[59]
Cu_0.2_Zn_0.4_Sn_0.4_	86	CO	− 0.4 (vs. RHE)	0.5 M NaHCO_3_	–	
Sn_56.3_Pb_43.7_	79.8	HCOO^−^	− 2.0 (vs. Ag/AgCl)	0.5 M KHCO_3_	45.7	[60]
PdSn/C	> 99	HCOOH	− 0.46 (vs. RHE)	0.5 M KHCO_3_	–	[20]
AgSn/SnO_*x*_	80	HCOOH	− 0.80 (vs. RHE)	0.5 M NaHCO_3_	16	[28]
SnO_*x*_/AgO_*x*_	> 95	HCOOH, CO	− 0.80 (vs. RHE)	0.1 M KHCO_3_	–	[69]
Urchin-like SnO_2_	62	HCOO^−^	− 1.0 V (vs. SHE)	0.5 M KHCO_3_	–	[71]
SnO_2_ Wire-in-tube	93	HCOOH, CO	− 0.89 to − 1.29 V (vs. RHE)	0.1 M KHCO_3_	–	[74]
SnO_2_ porous nanowires	80	HCOO^−^	− 0.80 (vs. RHE)	0.1 M KHCO_3_	–	[77]
Ultra-small SnO_2_ NPs	64	HCOO^−^	− 1.12 (vs. RHE)	1.0 M KHCO_3_	145	[78]
Ultra-small SnO	66	HCOO^−^	− 0.9 (vs. RHE)	0.5 M KHCO_3_	20	[35]
SnO_2_/graphene	93.6	HCOO^−^	− 1.8 (vs. RHE)	0.1 M NaHCO_3_	10.2	[70]
SnO_2_/CC	87 ± 2	HCOO^−^	− 1.6 (vs. Ag/AgCl)	0.5 M NaHCO_3_	45	[80]
Pd/SnO_2_ NS	55 ± 2	CH_3_OH	− 0.24 (vs. RHE)	0.1 M NaHCO_3_	–	[83]
TNS-2.0-SnO_2_	73	HCOOH	− 1.6 (vs. RHE)	0.1 M KHCO_3_	–	[84]
Cu, S Co-doped SnO_2_	58.5	HCOO^−^	− 0.75 (vs. RHE)	0.5 M NaHCO_3_	5.5	[88]
Sn(S)/Au	93.3	HCOO^−^	− 0.75 (vs. RHE)	0.1 M KHCO_3_	55	[92]
SnS_2_/RGO	84.5	HCOO^−^	− 1.4 (vs. Ag/AgCl)	0.5 M NaHCO_3_	13.9	[93]
SnS_2_ monolayers	94 ± 5	HCOO^−^	− 0.8 (vs. RHE)	0.1 M KHCO_3_	–	[96]
5%Ni–SnS_2_	93	CO, HCOO^−^	− 0.9 (vs. RHE)	0.1 M KHCO_3_	19.6	[97]

### Sn Monometallic Catalysts

Metallic Sn is the active form of the catalyst because it is the most thermodynamically stable form under electrocatalytic CO_2_ reduction conditions [37, 38]. Therefore, many studies on metallic Sn electrodes have been conducted to improve its catalytic performance.

Engineering the thickness, size, and morphology of a material has been regarded as popular and effective method to increase the catalytic performance of catalysts [39, 40]. For example, recent works have witnessed that the thickness of the catalyst layer has a great effect on the FE and current density for the conversion of CO_2_ to formate, which is related to the local proton concentration and electrical field when current density varies with layer thickness [19, 41]. Aside from the thickness, controlling the size and morphology of a catalyst is another effective way to tune catalytic activity [42]. The ratio of edge, corner, and plane sites can be adjusted by changing the morphology and size of the catalysts, so as to optimize the binding strength of intermediate ^·^COOH and ^·^CO during the CO_2_ER process [43, 44]. Kim et al. reported a new solar electrodeposition method to synthesize Sn catalysts with different morphologies, including rod, rectangular sheet, and dendrite structures, and then investigated their performance in the CO_2_ER [45]. Compared with other catalysts, the tiny rod-shaped Sn catalyst showed a high HCOOH formation rate with the maximum FE of 94.5% at 1.6 V (vs. Ag/AgCl). The results demonstrated that catalyst morphology plays a major role in formation rate and FE at various potentials.

**Fig. 3:**
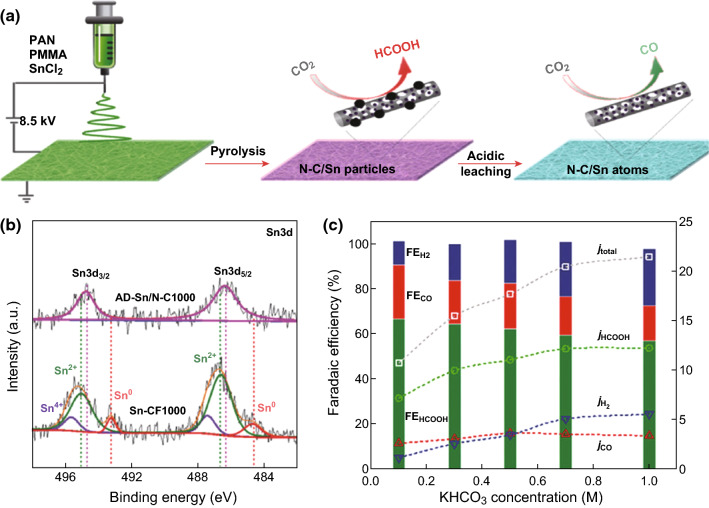
**a** Schematic illustration of the fabrication process of Sn-modified N-doped carbon nanofiber electrocatalysts. **b** High-resolution Sn3d XPS spectra for AD-Sn/N-C1000 and Sn-CF1000. **c** FEs and partial current densities of three products on Sn-CF1000 at − 0.8 V (vs. RHE) in CO_2_-saturated KHCO_3_ solution with different concentrations. Reprinted with permission from Ref. [21]

The poor dispersion of active inorganic materials leads to less active sites and low electroconductivity, resulting in a high overpotential, which considerably increases the energy cost [46]. For the CO_2_ER, the applied high voltage also accelerates the HER, thus suppressing the production of carbon compounds. Conventionally, they are loaded on electrically conducting carbon nanomaterials (e.g., carbon black, carbon nanotube, and graphene) to further improve their activities. Recently, such a support effect or interfacial interaction has been used to promote the CO_2_ER [21, 47, 48]. For example, Xie and coworkers constructed highly reactive Sn quantum sheets confined in graphene, which showed enhanced electrocatalytic activity and stability [28]. At a potential of − 1.8 V versus saturated calomel electrode (SCE), the Sn quantum sheets confined in graphene attained a maximum FE of 89%, and the value was larger than 85% during the long test period of 50 h. The graphene sheet-supported Sn nanosheets increased the electrochemically active surface area (ECSA) and enhanced the overall electronic conductivity and then promoted fast electron transfer to CO_2_ to form the CO_2_^· –^ radical anion intermediate, which plays a fundamental role in facilitating formate formation. The other works are expanded to another study of carbon-supported Sn catalyst; for example, Wallace et al. developed Sn-CF catalysts for the CO_2_ER via an electrospinning technique followed by pyrolysis (Fig. 3a, b) [21]. The selectivity of the dominant product could be tuned by changing the structure of the Sn species. The catalyst containing Sn NPs (Sn-CF1000) resulted in efficient formate formation with a high current density of 11 mA cm^−2^ and an FE of 62% at a moderate overpotential of 690 mV (Fig. 3c). The activity was a result of stronger electronic interactions between the abundant pyridinic-N in the carbon nanofibers and the anchored Sn NPs. After the Sn particles were removed via acidic leaching, the obtained catalyst (AD-Sn/N-C1000) had only abundant atomically dispersed Sn species, which promoted the conversion of CO_2_ to CO with a high FE of 91% at a low overpotential of 490 mV. Because of the abundance of pyridinic-N defects in carbon nanofibers, the Sn atoms in AD-Sn/N-C1000 might have coordinated with pyridinic-N, and the formed Sn–N moieties may have acted as new active sites for the CO_2_ER.

In addition to carbon materials, oxides have also been demonstrated as a kind of excellent support material to obtain hybrids for the CO_2_ER. Some metal oxides with restricted conductivity, high surface area, and large porosity like g-Al_2_O_3_ and ZSM5 also work as good substrates for catalysts. Basu et al. used the pore walls of g-Al_2_O_3_ and ZSM5 to support metallic Sn to overcome the disadvantage of non-conductive of substrates [49]. At a 20 wt% Sn catalyst loading, the Sn adhered to the porous wall of g-Al_2_O_3_ and ZSM5 in a monodispersed form without intermissions or agglomerates. In an electroreduction cell, the 20Sn/ZSM5 cathode produced a higher current of 190 mA cm^−2^ at − 2 V (vs. Ag/Ag^+^) and greater FE (20.4%) toward methane compared with the 20Sn/Al_2_O_3_ cathode (160 mA cm^−2^, 12.9%).

### Bimetallic or Multimetallic Sn Catalysts

In contrast to pure metal electrodes, alloy catalysts, which help to accurately control the surface electronic state and binding energy of electrocatalysts to optimize catalytic activity, are of particular interest [50]. This strategy has been widely used to optimize a range of electrocatalytic reactivity, such as the oxygen reduction reaction (ORR) and HER [51–53]. For the CO_2_ER, early studies have shown that the modification of foreign atoms on the metallic surface can alter the selectivity for CO or HCOO^−^ on the electrodes [18]. As Sakata et al. reported for Cu alloys with other metals, they found that alloying had a considerable effect on the onset potentials for CO_2_ electroreduction and that some alloys were able to create products that two separate metals could not produce [54]. Until now, a good deal of evidence has suggested that the combination of different types of metals to catalyze CO_2_ reduction affords the opportunity to better modulate the surface chemical environment and relative binding with different intermediates [55–57].

**Fig. 4:**
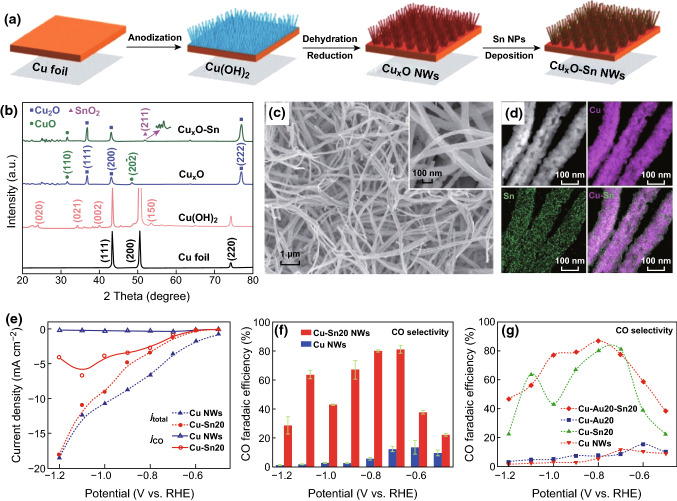
**a** Illustration of the fabrication process of Cu_*x*_O-Sn NWs. **b** XRD patterns of Cu foil, Cu(OH)_2_, Cu_*x*_O, and Cu_*x*_O–Sn. **c** Typical SEM images of Cu_*x*_O-Sn NWs. **d** STEM elemental mapping results of Cu_*x*_O–Sn NWs. **e** Linear sweep voltammetry (LSV) curves in 0.1 M KHCO_3_ solution for CO_2_ electroreduction on Cu NWs and Cu–Sn NWs. **f** Comparison of the FE of CO at different potentials between Cu NWs and Cu–Sn NWs. **g** Potential-dependent CO FE for Cu, Cu–Sn, Cu–Au, and Cu–Au–Sn NWs. Reprinted with permission from Ref. [63]

At present, many kinds of metals (e.g., Cu, Pd, Pb, Bi, etc.) have been chosen to combine with Sn to obtain binary or ternary alloys [58–62]. For example, a bimetallic Cu–Sn electrocatalyst was prepared through the electrodeposition of Sn species on the surface of oxide-derived copper, followed by annealing [58]. The introduction of Sn species changed the surface selectivity, with the result that the H binding sites were disturbed, inhibiting the evolution of H_2_ without altering the activity of CO_2_ reduction. The results showed that the Cu–Sn bimetallic surface exhibited highly selective and stable performance, resulting in better than 90% FE toward CO for long period of time (> 14 h) at − 0.6 V (vs. RHE). Recently, Wallace and coworkers have reported Sn NP-decorated copper oxide hybrid nanowire (NW) catalysts that were able to reduce CO_2_ to CO with an FE of 90% at a moderate overpotential of 0.69 V (Fig. 4a–f) [63]. The enhanced performance might have arisen from the synergistic interaction between the Sn NPs and Cu_*x*_O NWs, which was confirmed by changing the properties of the Cu_*x*_O NWs. If Sn NPs in the composite were replaced with Au NPs, Cu–Au NWs were formed, displayed an FE_CO_ similar to Cu NWs. However, the FE_CO_ changed dramatically after introducing Sn onto the Cu–Au NWs (Fig. 4g). It can be deduced that the introduction of Sn was the key to improving the CO selectivity. Cui and coworkers presented a thermodynamic analysis of the reaction energetics using density functional theory (DFT) calculations, which also suggested that Cu–Sn alloys could suppress the production of H_2_ and CO to achieve high formate selectivity. In the in situ X-ray absorption studies of Sn L_3_-edges and Cu K-edges, Sn has presented positive oxidation states in CuSn_3_ catalysts. The synthesized CuSn_3_ exhibited an FE of 95% toward formate at − 0.5 V (vs. RHE) and an excellent stability after 50 h in an initial study [64]. Moreover, Cu–Sn catalysts with a dendritic foam structure were also prepared for the CO_2_ conversion, showing excellent selectivity toward CO, and an FE for CO formation with a value up to 93–94% over a wide potential range [12]. In addition, better properties can be achieved by the incorporation of additional metal ions. For instance, Berlinguette and coworkers reported on Cu–Zn–Sn ternary alloys for CO_2_ electrocatalysis and showed that the addition of Sn suppresses H_2_ production in favor of CO or HCOOH production [59].

**Fig. 5:**
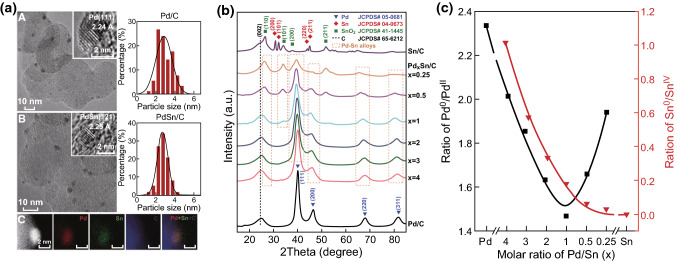
**a** TEM images and size distributions of the NPs in Pd/C and PdSn/C. **b** XRD patterns of the catalysts with different Sn contents. **c** Relative intensity ratios of Pd^0^/Pd^II^ and Sn^0^/Sn^IV^ versus the molar ratios of Pd/Sn. Reprinted with permission from Ref. [20]

The phase composition of the catalysts has great influence on activity and selectivity. To probe this, Ismail et al. fabricated Au–Sn bimetallic NPs with different intermetallic phases for direct use as a catalyst for the CO_2_ER. It was found that the formation of syngas and formate could be tuned by changing the composition of the intermetallic phase(s) efficiently. Selective isotopic labeling experiments have suggested that CO_2_ supplied through fast equilibrium with the bicarbonate on the electrode surface, which was proved by Raman spectroelectrochemistry. The results also showed the generation of formate anions on the AuSn phase at a notably less negative potential than on the pure Sn electrode [65]. Another research study by Chen’s group confirmed the close relationship between composition and properties [20]. They developed an activated carbon (AC)-supported Pd–Sn alloy NP electrocatalyst with varied Pd/Sn composition (Fig. 5a, b) and found a variation in the relative intensity ratios of Pd^0^/Pd^II^ and Sn^0^/Sn^IV^ with respect to the molar ratios of Pd/Sn in Pd, Sn, and alloyed Pd_*x*_Sn NPs (Fig. 5c). The authors found that the activity of HCOOH and CO, and the selectivity were highly dependent on the surface electronic structure of the alloy. The highest FE of nearly 100% for producing HCOOH was obtained over the PdSn/C catalyst at the lowest overpotential of − 0.26 V, where both CO formation and H_2_ evolution were completely suppressed. The changes in the HCOOH FE and the overpotential were synchronous with those of Pd^0^/Pd^II^ in Pd_*x*_Sn NPs, indicating that the activity for producing HCOOH is sensitive to the surface oxide species on alloyed NPs. DFT calculations suggested that the formation of the key reaction intermediate HCOO^·^, as well as the product formic acid, was the most favorable over the PdSn alloy catalyst surface with an atomic composition of PdSnO_2_, which is consistent with experimental findings.

### Sn Oxides

**Fig. 6:**
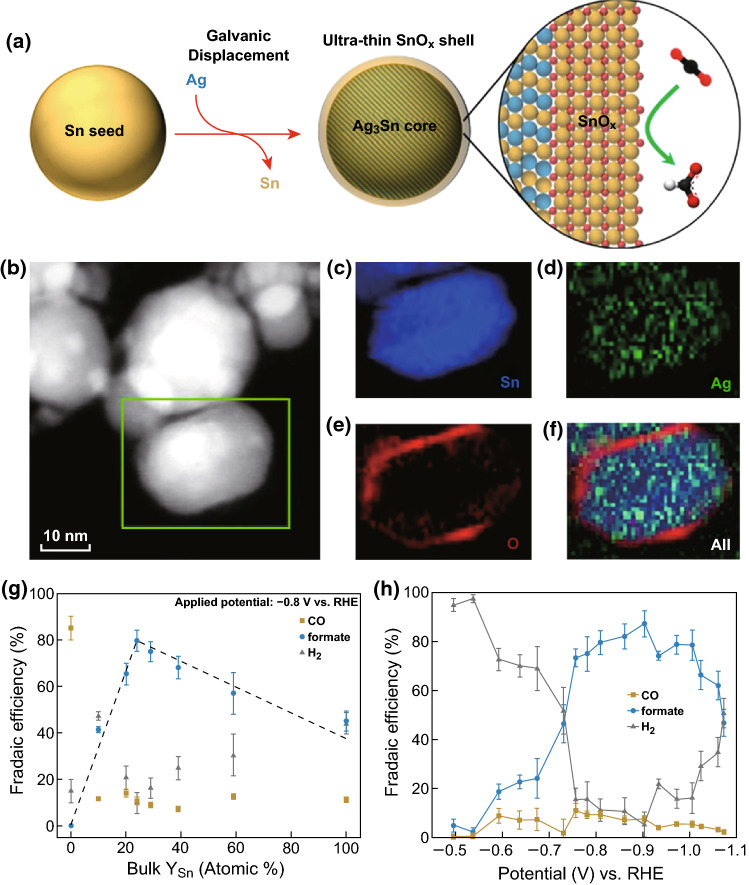
**a** Synthesis of AgSn/SnO_*x*_ core–shell catalysts. **b** HAADF-STEM image of Ag_76_Sn_24_. **c–f** EELS mapping of the selected region showing the elemental distribution of Sn, Ag, O, and their overlay. **g** CO_2_ reduction Faradaic efficiencies of AgSn/SnO_*x*_ catalysts with different Sn concentrations. **h** FE of Ag_76_Sn_24_ catalyst for CO, formate, and H_2_. Reprinted with permission from Ref. [28]

Many reported works have proved that an oxide layer with higher roughness and greater surface area on the surface of the metal would greatly influence the electrochemical process and is significant to the selectivity and activity of the electroconversion of CO_2_ [66, 67]. In the study of metallic Sn, a growing supply of experimental evidence, suggests the existence of surface oxides on Sn during the CO_2_ER, and it is a key factor in the formation of formate [29, 30, 68]. For example, in a recent work, Jiao’s group synthesized AgSn/SnO_*x*_ core–shell catalysts through a method of galvanically displacing Sn seeds with Ag, in which the AgSn bimetallic core influenced the high electronic conductivity, and an ultra-thin partially oxidized SnO_*x*_ shell was responsible for catalytic CO_2_ conversion (Fig. 6) [28]. They found a volcano-like relationship between the composition and the electrocatalytic performance, in which the optimal partially oxidized SnO_*x*_ shell thickness was ~ 1.7 nm. In the electrokinetic studies, the Tafel slope for CO_2_ conversion to formate was ~ 110 mV dec^−1^, which suggested that the RDS on AgSn/SnO_*x*_ core–shell catalysts was the first electron transfer. Moreover, DFT calculations showed that the SnO (101) surfaces with oxygen vacancies were active and stable at highly negative potentials, and crucial for CO_2_ activation. Similarly, relevant research for SnO_*x*_ film was also reported by Cuenya’s group through electrodepositing Sn on O_2_ plasma pre-oxidized Ag films to create SnO_*x*_/AgO_*x*_ catalysts. They proposed that the surface roughness and stable Sn^δ+^/Sn species in SnO_*x*_ showed enhanced activity and stable CO/HCOO^−^ selectivity [69]. In addition, Broekmann and coworkers utilized in operando Raman spectroscopy to monitor the oxidation state changes of SnO_2_ during the CO_2_ER [36]. They found that the efficiency of formate production was significantly decreased after the SnO_2_ was reduced to metallic Sn at very negative potentials. It gave powerful evidence that oxides can be used as a kind of efficient catalyst.

**Fig. 7:**
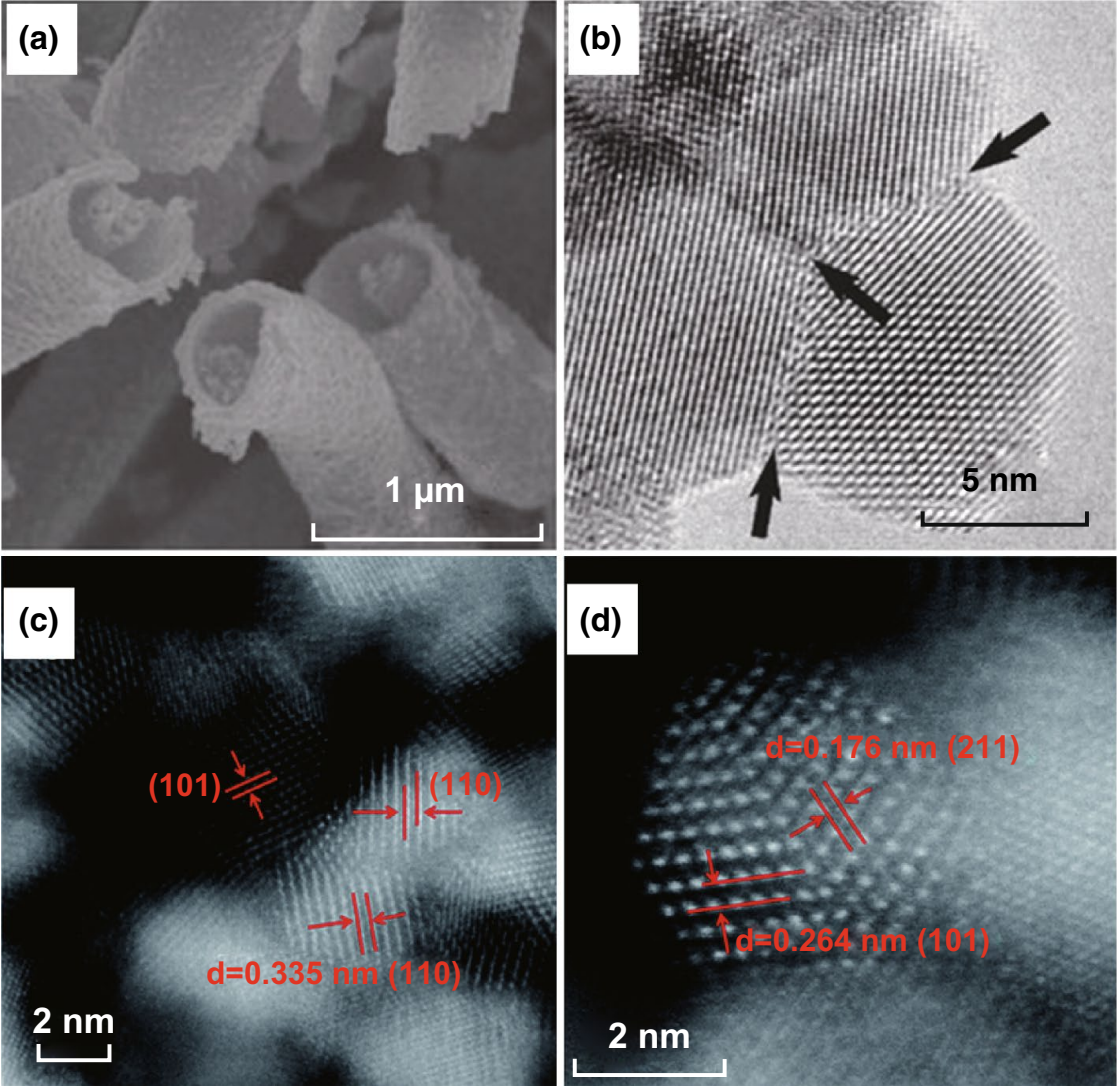
**a** SEM image of the WIT SnO_2_ nanofibers. **b** High-resolution TEM (HRTEM) image of the WIT SnO_2_ nanofibers with a high density of GBs. Reprinted with permission from Ref. [74]. **c, d** HRTEM images of SnO_2_ nanoparticles. Reprinted with permission from Ref. [78]

Following this trend, many studies on Sn-based oxides have been conducted to further improve electrocatalytic performance. Deliberately designing Sn-based oxides with controlled morphologies, structure, and chemical compositions are popular research topics at present. For example, Meyer et al. synthesized tin oxide nanocrystals with a high surface area and found that the FEs for formate production on Sn electrodes were varying with morphologies, and the maximum current efficiency reached on 5 nm tin oxide NPs [70]. In addition, urchin-like SnO_2_ [71], coralline-like SnO_2_ [72], and hierarchical SnO_2_ microsphere [73] catalysts have been synthesized, and they exhibited good catalytic activity toward CO_2_ electroreduction. Considering that a 1D structure possesses a high surface area and more edge sites, and can also facilitate charge transfer, Li’s group fabricated a 1D SnO_2_ with a wire-in-tube (WIT) structure via electrospinning and calcining at air, with a nanofiber that was composed of NPs interconnected through grain boundaries (GBs) (Fig. 7a, b) [74]. The WIT SnO_2_ nanofiber showed superior selectivity and stability for C_1_ products (HCOOH and CO), and the FE achieved was greater than 90%. The excellent catalysis activity may have resulted from the following aspects: (1) The BET analysis showed that the surface area of the WIT SnO_2_ nanofiber was 10 times that of the SnO_2_ NP, which may introduce more active sites for CO_2_^·−^ absorption. (2) The authors speculated that the field-induced reagent concentration (FIRC) effect might help stabilize the adsorbed CO_2_^·−^ intermediates, leading to superiority in the CO_2_ER to explain the catalysis activity enhancement. (3) The generation of high-density GBs could reform the bonding strengths between the adsorbate and the metal to stabilize the catalytically active surfaces [75, 76]. Recently, the dominant role of GBs in SnO_*x*_ for the CO_2_ER has been further explained by Spurgeon’s group and Ajayan’s group. They synthesized SnO_2_ porous nanowire catalysts (Sn-pNWs) and ultra-small SnO_2_ NPs with a high density of GBs (Fig. 7c, d), respectively [77, 78]. The authors confirmed that the structure rich in GBs would introduce new catalytic active sites to exhibit a higher energy conversion efficiency of CO_2_ to value-added chemicals than analogous catalysts.

Apart from SnO_2_, tin monoxide (SnO) has been also explored for CO_2_ reduction by Hu’s group [35]. They prepared SnO through the pyrolysis of SnC_2_O_4_/C precursor in N_2_ atmosphere. Ultra-small SnO NPs (2.6 nm) were completely reduced to Sn NPs of similar size and dispersion during the CO_2_ electroreduction. The derived catalyst exhibited a higher selectivity and a higher partial current density in CO formation than other Sn catalysts. The authors suggested that the high activity could be attributed to the ultra-small size of the SnO NPs, while the high selectivity could be attributed to a local pH effect arising from the dense packing of NPs in the conductive carbon black matrix.

**Fig. 8:**
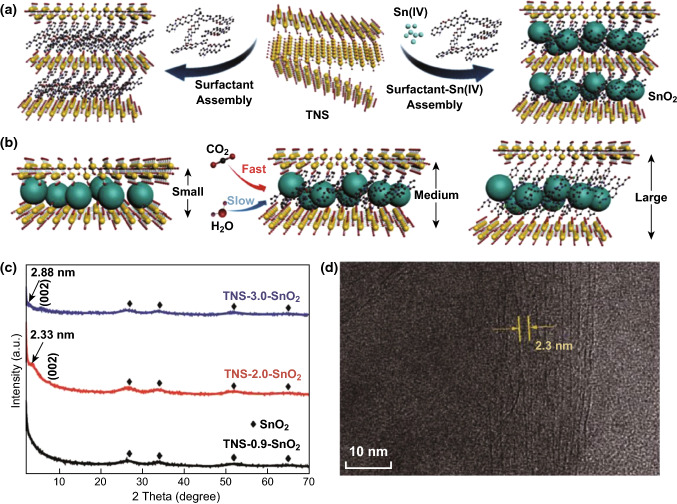
**a** Schematic illustration of the surfactant-induced 2D assembly of confined space with TNS and SnO_2_ nanoparticles. **b** Schematic illustration of 2D TNS-SnO_2_ assembly with different interlayer spacings for electrocatalytic CO_2_ reduction. **c** XRD patterns of TNS-0.9-SnO_2_, TNS-2.0-SnO_2_, and TNS-3.0-SnO_2_. **d** HRTEM of TNS-2.0-SnO_2_. Reprinted with permission from Ref. [84]

The combination of nanostructure engineering and hybridization are effective strategies that have been widely employed to improve electrocatalytic performance. Carbon material is undoubtedly the first choice of researchers for obtaining hybrid materials that can facilitate charge transfer [79]. Recently, Zhang’s group has fabricated a 3D hierarchical structure composed of mesoporous SnO_2_ nanosheets on carbon cloth (SnO_2_/CC) via a facile combination of hydrothermal reaction and calcination [80]. The as-prepared electrode showed high current density, high selectivity, and long-term stability at moderate overpotentials for the electroreduction of CO_2_ to formate in aqueous media. The superior performance of the SnO_2_/CC electrode was attributed to both the robust and highly porous hierarchical feature and the conductive CC as a 3D support and a current collector. Another effective strategy is the fabrication of metal/oxide interactions, which has been widely utilized to improve the kinetics for chemical catalysis. For instance, Wang et al. have reported on composition-dependent Cu/SnO_*x*_ NPs supported on a carbon nanotube (Cu/SnO_*x*_–CNT) catalyst. The productions changed with the composition of the catalysts [81]. Pd as a potential catalyst for CO_2_ER is very easily poisoned by CO, which is an important intermediate during the CO_2_ER [82]. This poison then inhibits further electroreduction of the ^·^CO intermediate. Recently, Zheng and coworkers have fabricated a 2D hierarchical structure comprised of ultra-thin Pd nanosheets partially capped by SnO_2_ NPs (Pd/SnO_2_) [83]. The authors took advantage of SnO_2_ to enhance the adsorption of CO_2_, but weakened the CO binding on Pd due to the as-built Pd–O–Sn interfaces, and enable multielectron transfer for selective electroreduction of CO_2_ into CH_3_OH as a major product. A maximum FE of 54.8 ± 2% for CH_3_OH was achieved at − 0.24 V (vs. RHE) in 0.1 M NaHCO_3_ solution. The selectivity of production is always a vexing question for CO_2_ER electrocatalysts. Zheng et al. constructed a 2D confined space as a molecular reactor, skillfully assembling the Sn (IV) ions into the interlayer spacing of neighboring atomically thin titanium nanosheets (TNSs), and further converting them into SnO_2_ NPs by hydrolysis (Fig. 8a) [84]. This hybrid structure effectively provided a hydrophobic environment and a confined space, which impeded the transfer of buffer electrolyte onto the SnO_2_ electrocatalyst surface, thus tuning different selectivities of CO_2_ER and HER (Fig. 8b). The interlayer spacing of lamella assemblies can vary from ~ 0.9 to 3.0 nm, and this was tailored by a variety of cationic surfactants. The varied interlayer spacing was also confirmed by XRD and HRTEM imaging (Fig. 8c, d). TNS-2.0-SnO_2_ with a medium interspace distance (~ 2.0 nm) showed an excellent FE of 73% for formate at − 1.6 V (vs. RHE). Moreover, the whole assembled structures and performances remained good after a lengthy time test.

It is well known that doping can change local structures and generate extrinsic defects, and thus improve reactivity and conductivity [85]. Doping other elements into the crystal lattice of metal oxide is another effective method for improving the electrochemical performance of electrocatalysts. Keith et al. predicted that doping Sn electrodes with Ti, V, Nb, or Zr would result in lower overpotentials for CO_2_ reduction compared with undoped tin oxide [86]. Mu’s group prepared nanosized fluorine-doped tin oxide (n-FTO) via direct chemical reaction between tin oxide powders and hydrofluoric acid at room temperature [87]. The n-FTO electrode exhibited good electrocatalytic ability for CO_2_ reduction under low potentials. Liang et al. prepared a series of Cu and S co-doped SnO_2_ materials through a facile hydrothermal method [88]. The elements Cu and S (Cu^2+^ in a 1:1 molar ratio with S^2−^ ions) were doped in SnO_2_ at a mole ratio of 1:10 and labeled SC_10_, which showed the optimum electrocatalytic activity for the reduction of CO_2_ to formate as compared with undoped SnO_2_. The overpotential examined was as low as 130 mV, and the maximum current density also increased to 5.5 mA cm^−2^ at − 1.2 V (vs. Ag/AgCl), which was 7 times higher than that of pure SnO_2_. The stability of the catalyst was maintained for more than 33 h, and the FE of formate was 58.5%.

### Sn Sulfides

As earth-abundant materials in nature, sulfide-derived metals have attracted tremendous attention for various applications due to their unique surface structures, local environments, and physicochemical characteristics of high electrical conductivity and good thermal stability, which enhances the kinetics of electron transfer and thus improves catalytic activity [89]. Sulfide-derived materials have been used as an effective way to optimize the activity and selectivity of CO_2_ reduction performance. For example, sulfur-modified Cu catalysts [90] and sulfur-doped indium catalysts [91] produce formate with high selectivity and high activity.

For Sn sulfides, sulfur-modulated tin (Sn(S)) catalysts were synthesized through the atomic layer deposition of SnS_*x*_ followed by a reduction process [92]. This was done by sputtering the Sn(S) film on Au needles (Sn(S)/Au) as the electrode for further electrochemical tests The Sn(S)/Au electrodes showed significantly better performance than the pure Sn NPs/Au samples, which reduced CO_2_ to formic acid with a FE of 93% at − 0.75 V (vs. RHE). Moreover, SnS_2_ with a unique layered structure has been reported to have outstanding properties for applications in the CO_2_ER. Li et al. synthesized a 2D SnS_2_/RGO composite for electrocatalytic reduction of CO_2_ to formate at an overpotential as low as 0.23 V, and a maximum FE of 84.5% was achieved at an overpotential of 0.68 V [93]. Atomic thickness facilitates the exposure of low-coordinated metal atoms on the surface and increases electron transport and mass diffusion, which provides more active sites, superior corrosion resistance, and high mechanical toughness in the electroreduction of CO_2_ [94, 95]. In a work published by the Luo’s group, SnS_2_ monolayers were synthesized by a facile Li intercalation/exfoliation method. The resulting catalyst exhibited efficient production of HCOO^−^ with a high FE of 94 ± 5% at − 0.8 V (vs. RHE) and excellent long-term durability (over 80 h) [96]. The XRD analysis of the catalysts after electrolysis indicated that the SnS_2_ monolayers were partially reduced to metallic Sn in the CO_2_ electroreduction. In sharp contrast, the bulk counterpart (SnS_2_ bulk) generated only a small amount of formate. Theoretical studies revealed that the atomic-scale thickness favored the key initial step for producing HCOO^·^ intermediates and the subsequent proton–electron transfer, leading to superior electrocatalytic performance for the production of formate from CO_2_.

**Fig. 9:**
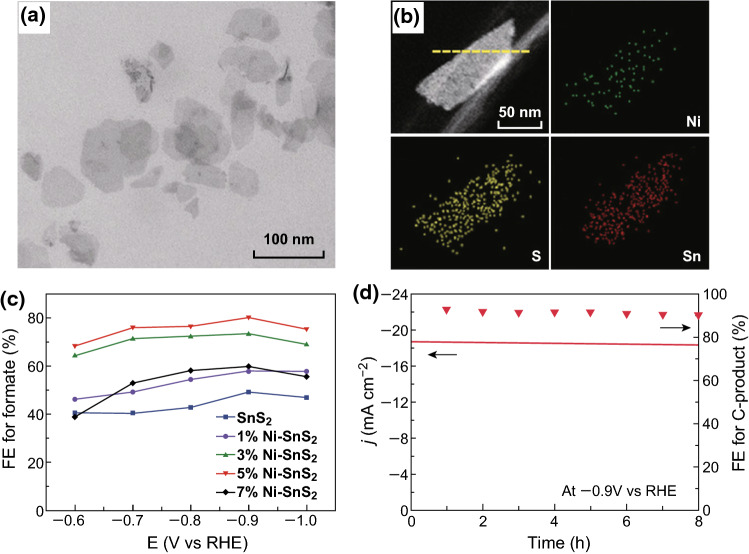
**a** TEM image of 5%Ni-SnS_2_ nanosheets. **b** HAADF-STEM and STEM-EDS elemental mapping images of individual 5%Ni-SnS_2_ nanosheets. **c** FEs for formate production over pristine SnS_2_ and Ni-doped SnS_2_ nanosheets. **d** Long-term stability performance of 5%Ni-SnS_2_ nanosheets at − 0.9 V (vs. RHE) for 8 h. Reprinted with permission from Ref. [97]

Typically, elemental doping is helpful not only to the band structure and charge redistribution, but also to adjusting the valence state of the active sites, resulting in good electrochemical performance. Zeng’s group modified atomically thin SnS_2_ nanosheets with Ni doping for enhanced performance in CO_2_ electroreduction (Fig. 9) [97]. The Ni-doped SnS_2_ nanosheets exhibit a dramatic increase in current density and FE for a carbonaceous product than those of the pristine SnS_2_ nanosheets. When the Ni content is 5 at% (5% Ni-SnS_2_), the catalysts achieve an excellent FE of 93% for carbonaceous products, with a considerable current density of 19.6 mA cm^−2^ at − 0.9 V (vs. RHE). Moreover, the 5% Ni-SnS_2_ nanosheets maintained high stability for FE without great decay of current density during the potentiostatic test. The mechanistic study showed that the Ni doping increased the defect level and reduced the work function of the SnS_2_ nanosheets, which resulted in promoting CO_2_ activation and further improving performance in CO_2_ electroreduction.

## Conclusions and Outlook

In this review, we have briefly summarized several major kinds of Sn-based materials in terms of synthesis, the effect of structure and composition factors on the performance, as well as key factors for applications in CO_2_ electroreduction. Until now, a variety of Sn-based catalysts have been developed to convert CO_2_ to valuable products. Metallic Sn is an active form and the most stable form of these Sn-based catalysts; however, only a few strategies have been used to tune the performances of metallic Sn catalysts, such as by modifying their thickness, size, and morphology. Sn alloy catalysts are also effective catalysts for the CO_2_ER. Their catalytic activity and selectivity is highly depended on the kinds of metals and phase composition, while the accurately controlled preparation of Sn alloy catalysts is challenging work. During the study of metallic Sn catalysts, it was found that the existence of surface oxides on Sn is of benefit to the formation of formate; therefore, Sn oxides with different morphologies, structures, and chemical compositions were prepared. As earth-abundant materials in nature, sulfide-derived Sn sulfides have been applied as catalysts for CO_2_ reduction with high activity and selectivity. Currently, significant progress has been made in the area of Sn-based catalysts for the CO_2_ER. However, from the perspective of the catalysts, there are still several obstacles to overcome before nanostructured Sn-based catalysts are widely employed in practical and commercial applications of the CO_2_ER. To achieve this goal, the following challenges related to Sn-based catalyst development for the CO_2_ER should be focused on:

1. Low catalytic activity, selectivity, and durability. Generally, a large overpotential is inevitable, which stems from the formation of the key intermediate CO_2_^·−^, multiple electron or proton coupling processes, and different reaction pathways, and as a result, catalysts always exhibit low catalytic activity and efficiency. In addition, the competition between CO_2_ reduction and HER always exists. Thus, the products obtained at a fixed overpotential are usually a mixture rather than a single product, and poor product selectivity is one of the biggest bugbears during the CO_2_ER for Sn-based and other kinds of catalysts. Meanwhile, the changes in structure and composition, and the deposition of inert by-products on the catalyst surface after a long reaction time lead to the inactivation of the catalyst. As a consequence, activity degradation is also a severe problem due to the instability of Sn-based catalysts. Therefore, the preparation of catalysts and the development of new technologies to enhance catalytic efficiency and product selectivity, and to prolong the catalyst lifetime are goals to strive for.
2. Insufficient fundamental understanding and standard experimental systems. Although the underpinning mechanisms of Sn-based catalysts have been studied extensively, the underlying fundamental processes are still not well understood at the molecular level. DFT is a powerful tool for understanding the intermediates and active species in the reaction. However, with the increasing complexity of catalyst components, the identification of actual catalytic active sites will become more difficult. A combination of in situ, ex situ, and operando studies on the model catalysts with computational strategies is an effective means to find insight into the electrochemical reaction mechanisms involved at the molecular level. This strategy will provide a way to design and find high-performance electrocatalysts for the CO_2_ER. In addition, the test systems and the operating environments in the literature are different, which is not conducive to the mutual evaluation and comparison of different experimental cases.

The development of Sn-based catalysts could have breakthroughs related to the following aspects:

1. Manipulation of surface structure and defects of the catalysts. Improving the surface properties of nanomaterials is an effective strategy for finding high-efficiency and stable electrocatalysts. In the surface properties of catalysts, defects exist widely in most materials, which may furnish unexpected physical and chemical properties through the modulation of catalyst electronic properties. Therefore, it is helpful to enhance electrocatalytic performance. In recent years, despite the remarkable progress in Sn-based catalysts, the studies on defects are still scarce. Defect engineering in Sn-based nanostructured catalysts has provided an exciting opportunity to improve the performance of the CO_2_ER. Realizing the controllable synthesis of defects and making clear what effects the defect type and concentration have on electrocatalytic performance provide the opportunity to further improve the performance of Sn-based catalysts.
2. Single-atom catalysts with fully exposed, highly selective, and well-defined active sites have shown great potential for tackling the above challenges in the CO_2_ER. For example, atomically dispersed Ni, Fe, and Co coordinated with nitrogen atoms in carbon substrates have demonstrated impressive activity for the CO_2_ER. Based on this, more attention should be paid to Sn–N–C materials, which could be ideal catalysts for CO_2_ reduction.

In conclusion, we have summarized recent progress on the Sn-based catalysts for the CO_2_ER. In spite of tremendous challenges facing this field for large-scale applications, including low performance, unsatisfactory produce yield, and high energy consumption, it is believed that by continuously optimizing the catalysts and measurement systems, the dream of obtaining high-yield useful fuels/chemicals from CO_2_ reduction will be true in the future.
